# Comparison of Next-Generation Sequencing Technologies for Comprehensive Assessment of Full-Length Hepatitis C Viral Genomes

**DOI:** 10.1128/JCM.00330-16

**Published:** 2016-09-23

**Authors:** Emma Thomson, Camilla L. C. Ip, Anjna Badhan, Mette T. Christiansen, Walt Adamson, M. Azim Ansari, David Bibby, Judith Breuer, Anthony Brown, Rory Bowden, Josie Bryant, David Bonsall, Ana Da Silva Filipe, Chris Hinds, Emma Hudson, Paul Klenerman, Kieren Lythgow, Jean L. Mbisa, John McLauchlan, Richard Myers, Paolo Piazza, Sunando Roy, Amy Trebes, Vattipally B. Sreenu, Jeroen Witteveldt, STOP-HCV Consortium, Eleanor Barnes, Peter Simmonds

**Affiliations:** a MRC-University of Glasgow Centre for Virus Research, Glasgow, United Kingdom; b Oxford Genomics Centre, Wellcome Trust Centre for Human Genetics, University of Oxford, Oxford, United Kingdom; c Nuffield Department of Medicine, University of Oxford, Oxford, United Kingdom; d Virus Reference Department, Public Health England, London, United Kingdom; e University College London (UCL), Division of Infection and Immunity, London, United Kingdom; f Roslin Institute, University of Edinburgh, Edinburgh, United Kingdom; University of Texas Medical Branch

## Abstract

Affordable next-generation sequencing (NGS) technologies for hepatitis C virus (HCV) may potentially identify both viral genotype and resistance genetic motifs in the era of directly acting antiviral (DAA) therapies. This study compared the ability of high-throughput NGS methods to generate full-length, deep, HCV sequence data sets and evaluated their utility for diagnostics and clinical assessment. NGS methods using (i) unselected HCV RNA (metagenomics), (ii) preenrichment of HCV RNA by probe capture, and (iii) HCV preamplification by PCR implemented in four United Kingdom centers were compared. Metrics of sequence coverage and depth, quasispecies diversity, and detection of DAA resistance-associated variants (RAVs), mixed HCV genotypes, and other coinfections were compared using a panel of samples with different viral loads, genotypes, and mixed HCV genotypes/subtypes [geno(sub)types]. Each NGS method generated near-complete genome sequences from more than 90% of samples. Enrichment methods and PCR preamplification generated greater sequence depth and were more effective for samples with low viral loads. All NGS methodologies accurately identified mixed HCV genotype infections. Consensus sequences generated by different NGS methods were generally concordant, and majority RAVs were consistently detected. However, methods differed in their ability to detect minor populations of RAVs. Metagenomic methods identified human pegivirus coinfections. NGS provided a rapid, inexpensive method for generating whole HCV genomes to define infecting genotypes, RAVs, comprehensive viral strain analysis, and quasispecies diversity. Enrichment methods are particularly suited for high-throughput analysis while providing the genotype and information on potential DAA resistance.

## INTRODUCTION

Hepatitis C virus (HCV) chronically infects more than 150 million people globally and is associated with the development of liver fibrosis, cirrhosis, hepatic failure, and hepatocellular cancer (1). Historically, treatment of HCV has been based on interferon alpha (IFN-α) and ribavirin (RBV), which are associated with high treatment failure rates and severe side effects. New all-oral directly acting antivirals (DAAs) with high efficacy rates and an improved safety profile have recently revolutionized the treatment of HCV. Most recently, oral therapies that target NS3, NS5A, and NS5B HCV proteins have been approved by the Food and Drug Administration and European Medicines Agency regulatory bodies (2, 3) and, used in combination, these DAAs achieve high sustained virological response (SVR) rates with minimal side effects (4). HCV is currently classified into seven major genotypes and 67 subtypes (5). At present, there is no truly pan-genotypic DAA treatment regimen with both drug choice and treatment duration defined by the viral genotype. Genotype 3 in particular appears less susceptible to DAA therapies (6). Therefore, the accurate assignment of viral genotype and subtype remains an important stratification parameter both in clinical trials of DAA therapy and in clinical practice.

Although a minority of patients fail to achieve SVR with all-oral combination therapy, failure more commonly occurs in patients with advanced liver disease, and optimal retreatment strategies in all patients who fail DAA therapies are currently unclear. Initially, it was reported that treatment failure with combination DAAs was rarely associated with the development of viral resistance-associated variants (RAVs), and therefore, the role for the development of sequencing technologies or phenotypic characterization to assess RAVs was unclear. However, with the exception of the NS5B inhibitors, each of the DAAs is known to have a low genetic barrier for the development of antiviral resistance, and naturally occurring HCV polymorphisms may confer DAA resistance. Currently, prescreening for RAVs prior to treatment is recommended only for the NS3 protease inhibitor simeprevir (7), since the Q80K mutation that can confer resistance is widely distributed among genotype 1a variants. However, while simeprevir may soon become obsolete in HCV treatment strategies, careful analysis of viral sequences by independent investigators has revealed that RAVs may emerge in association with DAA treatment failure even with the high barrier to resistance NS5B inhibitors (8). The emergence of resistance to DAAs targeting NS5A is clearly documented and of particular concern as these do not incur a significant fitness cost for replication. They can persist and transmit in the community (9).

Currently, the assessment of viral genotype commonly uses probe-based assays that target the highly conserved 5′ untranslated region (5′UTR), while the detection of RAVs currently relies upon the targeted analysis of genomic regions that rely on PCR Sanger sequencing; the application of this method is limited by problems with primer design for highly divergent HCV genotypes, genome coverage, and a restricted and inconsistent ability to detect both minor populations of RAVs as well as mixed-genotype/subtype [geno(sub)type] infections that may be relevant for treatment response. We therefore developed and compared next-generation sequencing (NGS) technologies for the generation of full-length HCV sequences, with the potential to accurately define HCV geno(sub)type while also simultaneously identifying both RAV and minor variant populations across the entire genome. Whole-genome sequencing (WGS) that could be routinely applied in clinical practice could inform retreatment strategies and also provide more-detailed sequence data to examine transmission events between individuals and potentially inform public health intervention strategies. Together, these capabilities would represent a major advance in the field.

We evaluated and compared three approaches across four United Kingdom laboratories in order to establish the robustness of pipelines for sequencing HCV RNA from plasma. The simplest, “metagenomic” approach obtains data that is unbiased by infecting genotype, with the potential benefit of detecting additional pathogens but with the substantial disadvantage that the vast majority of sequence reads obtained are of human origin and are discarded. “Enrichment” approaches provide an alternative in which HCV sequences are targeted for capture from metagenomic sequencing libraries using panels of oligonucleotide probes but at the expense of missing nontargeted pathogens and, potentially, divergent HCV sequences. Both techniques were compared with an approach in which the HCV genome is spanned by six overlapping PCR amplicons which are pooled and sequenced to a high depth by NGS.

In evaluating the effectiveness of metagenomic, enrichment, and PCR amplification approaches to HCV whole-genome sequencing, we compared data generated using a variety of protocols at different laboratory sites and so explored the reproducibility of aspects of the sequence data in independent trials, including the generation of accurate consensus sequences, detection of quasispecies diversity, and full sequence coverage of the HCV genome. The analysis allowed us to define a relationship between sequencing depth and coverage with RNA viral loads and so predict the expected success rates for clinical samples. Finally, we explored the reproducibility of recovery of virus subpopulations and minor variants, using panels of mixed samples and DAA-associated polymorphisms.

## MATERIALS AND METHODS

### Samples.

A range of plasma samples, assay controls, and *in vitro* transcripts were used to evaluate NGS methods. These samples included the following.

### (i) Plasma samples.

Plasma samples from individuals infected with HCV genotypes 1a, 1b, 2, 3, and 4 were obtained from the HCV Research UK Biobank (http://www.hcvresearchuk.org/). Samples were used with informed consent conforming to the ethical guidelines of the 1975 Declaration of Helsinki, and study protocols were approved by the National Research Ethics Service (NRES) Committee East Midlands (reference 11/EM/0323). Viral loads (VLs) were measured by COBAS TaqMan PCR (Roche) according to the manufacturer's protocol. Viral loads were expressed as international units (IU) per milliliter.

### (ii) Mixed-genotype plasma samples.

Two samples from the United Kingdom Quality Control for Molecular Diagnostics (QCMD) HCV genotype panel containing mixed genotypes (HCVG10-02 [genotypes 1b and 3a] and HCVG10-04 [genotypes 3a and 5a]) were used. Artificial mixtures of plasma samples from HCV Research UK containing different geno(sub)types were created in defined ratios using viral loads measured by COBAS TaqMan PCR (see Table S1B in the supplemental material).

### (iii) *In vitro* RNA transcripts.

Full-length cDNA clones of the HCV strains H77 and JFH-1 were linearized with XbaI, treated with mung bean nuclease (New England BioLabs) to remove 5′-end overhangs and purified (PureLink PCR purification kit; Invitrogen). One microgram of linearized DNA template was used for RNA transcription using T7 RNA polymerase (MEGAscript; Ambion) for 1 h at 37°C. RNA was cleaned up using the RNeasy kit (Qiagen), and the integrity of the RNA was analyzed by nondenaturing agarose gel electrophoresis. RNA concentrations were determined using spectrophotometry. Transcripts were diluted in Tris-EDTA (TE) buffer and mixed in ratios at known concentrations before distribution to the four laboratories (see Table S1B in the supplemental material).

Together these samples were used to create the NGS evaluation panel. This panel comprised the following: (i) plasma samples from 27 individuals infected with single genotypes as determined by the referring laboratories and a negative control (see Table S1A in the supplemental material); (ii) seven samples containing a mixture of two samples of known genotypes spanning genotypes 1, 2, 3, and 4 (Table S1B); (iii) nine samples containing RNA transcripts from genotype 1a and 2a in ratios corresponding to 5,000:1 to 1:5,000 of genotype 1a and 2a *in vitro* RNA transcripts (IVTs), respectively.

### Sequencing methods. (i) Summary.

The combined evaluation panel of 43 samples was used to evaluate the performance of seven sequencing methods developed at four expert centers in the United Kingdom. The four expert centers were Glasgow (G), Oxford (O), Public Health England (PHE [P]), and University College London (UCL [U]) and are indicated by the letters before the method. Each sample was assayed in a blind manner using NGS platforms and either unselected (metagenomic) approaches (G-Meta and O-Meta), probe-based HCV sequence enrichment (G-SSel, G-Nimb, O-Capt, and U-Capt), or HCV-specific PCR amplification and sequencing (P-PCR) (methods and method codes given in Table 1). Data from each method were processed using analysis pipelines established at each center (Table 1). Processing of read data consensus sequence construction, assessment of genome coverage, and accuracy and quantification of quasispecies diversity used a common set of tools in an additional center that coordinated the analysis (Edinburgh, United Kingdom).

**TABLE 1:** Sequencing methods and analysis pipelines evaluated at each sequencing center in the United Kingdom

Center	Method	Method code	Sequencing method	Analysis strategy
Oxford	Metagenomic	O-Meta	Illumina RNA-Seq of total plasma RNA	Bespoke bioinformatic pipeline to infer metagenomic, consensus, and subpopulation level information^*a*^
	IDT	O-Capt	Genotype-specific HCV capture using IDT probes, followed by Illumina RNA-Seq^*a*^	Bespoke bioinformatic pipeline to infer metagenomic, consensus, and subpopulation level information^*a*^
Glasgow	Metagenomic	G-Meta	Illumina RNA-Seq of total plasma RNA	FastQC, Tanoti,^*b*^ in-house resistance mutation tools, *de novo* assembly using MetAmos
	SureSelect	G-Ssel	Genotype-specific HCV capture using SureSelect DNA probes, followed by Illumina RNA-Seq	FastQC, Tanoti, in-house resistance mutation tools, *de novo* assembly using MetAmos
	NimbleGen	G-Nimb	Genotype-specific HCV capture using NimbleGen RNA probes, followed by Illumina RNA-Seq	FastQC, Tanoti, in-house resistance mutation tools, *de novo* assembly using MetAmos
UCL	SureSelect	U-Capt	SureSelect^XT^ Target Enrichment library preparation and hybridization and enrichment using custom designed RNA probes, followed by Illumina DNA-Seq	Genome mapping,^*c*^ assembly and finishing using CLC Genomics Workbench from Qiagen. DAA analysis using in-house script
PHE	Pre-PCR	P-PCR	Genotype-specific nested PCR of 5 or 6 overlapping fragments, followed by Illumina sequencing.	Contig assembly by SPAdes 3.5.0. HCV contigs longer than 250-nt assembled and PCR fragments combined using Sequencher 5.0. Reads were remapped to assembled sequences using BWA 0.7.5.

a See reference 10.

b V. Sreenu, G. Nikolov, S. Alotaibi, T. Abdelrahman, K. Brunker, R. Orton, T. Klymenko, G. Wilkie, and E. Thomson, submitted for publication.

c Adapted from reference 17.

### (ii) O-Meta and O-Capt.

Total RNA was extracted from 500 μl plasma using the NucliSENS magnetic extraction system (bioMérieux) and eluted into 30 μl of kit buffer. Metagenomic libraries were prepared using the NEBNext Ultra Directional RNA Library Prep kit for Illumina (New England BioLabs); 5 μl (maximum, 10 ng) of RNA was fragmented (5 or 12 min at 94°C), reverse transcribed, amplified (5 to 18 PCR cycles) using indexed primers, and then purified into 0.85× volume Ampure XP (Beckman Coulter). Libraries were quantified (Quant-iT PicoGreen dsDNA [double-stranded DNA] assay kit; Invitrogen) and assessed for purity (TapeStation with D1K High Sensitivity kit; Agilent) before pooling in equimolar proportions and final normalization (KAPA SYBR Fast qPCR [quantitative PCR] kit; KAPA Biosystems). Metagenomic virus RNA sequencing (RNA-Seq) libraries were sequenced with 100-base paired-end (PE) reads on the Illumina HiSeq 2500 sequencing system with v3 rapid chemistry.

For capture, a 500-ng aliquot of the pooled O-Meta library was enriched using equimolar, pooled 120-nucleotide (nt) DNA oligonucleotide probes (10) using the xGen Lockdown protocol from Integrated DNA Technologies (IDT). Enriched pools were reamplified (12 cycles on-bead PCR), repurified, and normalized using qPCR, and 100-base PE reads were sequenced on a single run of the Illumina MiSeq system (v2 chemistry).

### (iii) G-Meta and G-Capt.

RNA was extracted from 200 μl plasma using the Agencourt RNAdvance blood kit (Beckman Coulter) eluted into 11 μl of water and then reverse transcribed using Superscript III (Invitrogen) with random hexamers and a NEB Second Strand Synthesis kit (New England BioLabs) for library preparation using the KAPA Library Prep kit (KAPA Biosystems) with index tagging by 16 cycles of PCR using KAPA HiFi HotStart (KAPA Biosystems) and NEBNext Multiplex Oligos (oligonucleotides) for Illumina Index Primer Sets 1 and 2 (New England BioLabs). Libraries were quantified by Qubit (ThermoFisher) and TapeStation (Agilent) and pooled at equimolar concentrations for sequencing on the Illumina MiSeq platform (v3 chemistry).

For capture, pooled G_meta libraries were enriched by either the NimbleGen SeqCap EZ system (Roche) (G_Nimb) or the SureSelect Target Enrichment system (Agilent) (G_SSel), the latter with double-scale reactions and hybridization for 36 h rather than the recommended 16 to 24 h, and then sequenced on the Illumina MiSeq platform using v3 chemistry (Illumina).

### (iv) U-Capt.

RNA was extracted (QIAamp viral RNA minikit [catalog no. 52904; Qiagen]) from 140 μl of plasma eluted into 60 μl of AVE buffer, RNA was concentrated to 10 μl by using either a Speedy-vac at 65°C or RNeasy MinElute Cleanup kit before first-strand cDNA synthesis (Superscript III reverse transcriptase kit; Life Technologies).

Second-strand cDNA synthesis used 20 μl from first-stand synthesis (Second Strand cDNA synthesis kit; NEB). SureSelect^XT^ Target Enrichment (Agilent) was used for library preparation, hybridization, and enrichment. A total of 120-mer RNA baits spanning 953 GenBank HCV reference genomes were designed by the PATHSEEK consortium and synthesized by Agilent Technologies. Purified double-stranded cDNA (ds cDNA) was quantified (Qubit dsDNA HS assay kit; Life Technologies) and sheared (200 to 500 ng ds cDNA for 150 s using Covaris E220 focused ultrasonication system). Samples containing <200 ng were bulked with human genomic DNA (gDNA) (Promega) prior to shearing. End repair, adapter ligation, hybridization, PCR pre- and postcapture, and all postreaction cleanup steps were performed according to the SureSelect^XT^ Automated Target Enrichment for Illumina Paired-End Multiplexed Sequencing 200 ng protocol (version F.2) on the Bravo platform WorkStation B from Agilent Technologies. All recommended quality control steps were performed on the 2200 TapeStation (Agilent Technologies). The samples were sequenced on an Illumina MiSeq sequencing platform with 500-bp v2 reagent sets. Base calling, adapter trimming, and sample demultiplexing were generated as standard producing paired FASTQ files for each sample.

### (v) P-PCR.

Viral RNA was extracted from 200 μl plasma (Qiagen Ultra Sens extraction kit). HCV genotype was defined using a pan-genotypic sequencing assay of the NS5B region as previously described (11). Whole-genome sequencing used HCV genotype-specific primers in five or six overlapping amplicons for each genotype/subtype (see Table S2 in the supplemental material). Viral RNA was amplified (single-step reverse transcription-PCR [RT-PCR]; Superscript III reverse transcriptase [Invitrogen]), followed by nested or seminested PCR. PCR products were purified (QIAquick kit; Qiagen) and quantified (Qubit dsDNA Broad Range and High Sensitivity Assay kits and the Qubit 2.0 fluorometer; Life Technologies). Alternate amplicons were pooled in two reaction mixtures of equimolar amounts, and 1 ng/μl of the pooled DNA was used for library preparation (Nextera XT DNA sample preparation kit; Illumina) according to the manufacturer's instructions. Indexed libraries were sequenced using Illumina MiSeq deep sequencing reagent kit v2 (Illumina).

For quality assurance of primers used for amplification, the primers were frequently validated by checking alignments of all publicly available genome sequences to detect any intragenotype variations; new batches of primers are validated side by side with old primer stock on samples that had been previously amplified and sequenced. Primer stocks are also revalidated every 6 months.

Several measures were in place to prevent and monitor PCR contamination, including the following: (i) inclusion of negative controls within each batch of extractions and amplifications; (ii) standard PCR workflows, such as directional material flow, geographical and temporal separation of PCR stages, reagent aliquoting, etc.; (iii) bioinformatic pipeline that includes the use of a depth threshold of >100 (as contaminants rarely have a depth greater than 10); and (iv) phylogenetic-tree-based contamination checking that includes all sequences within a run and those processed on several previously immediate runs.

### Bioinformatic processing. (i) Oxford.

As described previously (10, 12), low-quality bases were trimmed from demultiplexed sequences using QUASR v7.01 (www.bioconductor.org/packages/release/bioc/html/QuasR.html), and adapter sequences were removed using CutAdapt v1.7.1 (http://cutadapt.readthedocs.io/en/stable/index.html). Human sequences were excluded by mapping to the HG19 human reference genomes with Bowtie v2.2.4 (http://bowtie-bio.sourceforge.net/index.shtml), and HCV-derived reads were aligned to a local BLAST database of 165 HCV genomes collated by the ICTV (International Committee on the Taxonomy of Viruses). PE reads were assembled *de novo* into contiguous whole-genome sequences with Vicuna v1.3 and finished with V-FAT v1.0 (http://www.broadinstitute.org/scientific-community/science/projects/viral-genomics/v-fat). Reads were mapped back to the assembly using Mosaik v2.2.28 (http://gkno.me/pipelines.html#mosaik), and variants were called by V-Phaser v2.0 (http://www.broadinstitute.org/scientific-community/science/projects/viral-genomics/v-phaser-2).

### (ii) Glasgow.

Fastq file quality was assessed using FastQC (http://www.bioinformatics.babraham.ac.uk/projects/fastqc/). Sam files were created by mapping against 64 whole-genome HCV reference sequences using Tanoti (http://bioinformatics.cvr.ac.uk/tanoti.php) and *de novo* assembly using the MetAmos pipeline (http://www.cbcb.umd.edu/software/metamos/). Assemblies were viewed using UGene (http://ugene.net/). Genotype ratios were calculated by a kmer-based approach using kmers unique to each genotype.

### (iii) UCL.

Genome mapping, assembly, and finishing were performed using CLC Genomics Workbench (Qiagen version 7.5/7.5.1). All read pairs were subject to quality control, and reads were quality trimmed on the basis of a cutoff average Phred score of 30 and the presence of ambiguous nucleotides. Adapter trimming of Illumina-specific adapters was performed on all samples. Trimmed reads were mapped against a GenBank reference list containing 953 HCV genomes to identify the best matching HCV reference. Each sample was mapped using the default affine gap cost parameters followed by local realignment. Total base counts at each genomic position were recorded using an in-house script.

### (iv) PHE.

A subset of the MiSeq PE reads from each FATSQ file was compared to a local database of 1,684 HCV whole-genome reference sequences using BLAST to identify an optimum reference sequence for mapping and BWA-MEM (v0.7.5) (https://www.msi.umn.edu/sw/bwa). Utilizing SAMtools (http://samtools.sourceforge.net/mpileup.shtml), the resulting files were converted into BAM format. In-house software (QuasiBAM) generated consensus sequences for minority variants. Procedures were automated using a computational pipeline developed in-house with Python and C++. For detection of multiple HCV genotypes, FASTQ files derived from amplification of the NS5B genotyping fragment were digitally normalized (Kmer software) to reduce the number of duplicate reads and assembled (SPAdes v3.5.0; http://bioinf.spbau.ru/spades). Contigs were compared with a database of NS5B fragments representing different HCV genotypes using BLAST and stitched together to give the longest possible sequence from each genotype detected by the contig BLAST process. Where multiple genotypes were detected, the contigs were trimmed to match the length of the shortest sequence. The total population of reads (nonnormalized FASTQ files) were then reference mapped against the genotype-specific assembly contigs using BWA, and the proportion of reads mapping to each genotype was calculated using the statistics programs in the BamTools suite.

### (v) Consensus sequence generation.

For all methods, a majority base consensus sequence was calculated at each nucleotide site possessing 10 or more base reads. A global consensus sequence was generated similarly as a majority consensus sequence for the seven different sequencing methods. Any assembled sequence that was >5% divergent from those generated by other NGS methods were discarded.

### PCR amplicon sequencing.

A genotype 1-specific PCR was used to amplify sequences in the NS3 and NS5B regions (positions 3288 to 5727 and 7407 to 9366, respectively; total 4,100 bases) from genotype 1a and 1b panel members (n-12). Sanger sequencing used the dideoxy ABI sequencing systems in both directions using overlapping internal primers (see Table S2 in the supplemental material). Sequences were analyzed using Sequencher software (Gene Codes) and aligned using subtype-specific consensus sequences.

Sequences obtained from each method were compared with those derived from NGS methods (global consensus), and the numbers of nucleotide and amino acid sequence differences were recorded using the program Sequence Dist in the SSE package.

### RAV analysis.

For the RAV analysis, positions of interest were identified in the GenBank reference hepatitis C strain H77 polyprotein gene, complete coding sequence (AF011751). Each reference used for mapping was aligned to the HCV strain H77 reference to standardize the positions of interest, and the counts for each base were identified at the DAA-associated positions.

### Statistics.

Spearman's rank order correlation test was used to test the significance of the association between viral load and HCV read counts. The Kruskal-Wallis nonparametric one-way test of variance was used to compare detection and assembly of genotype 1/non-genotype 1 HCV reads. A *P* value of <0.05 was considered significant.

### Accession numbers.

HCV-specific reads for the 43 samples have been submitted to the European Nucleotide Archive (ENA) under projects PRJEB11791 (Oxford). Consensus nucleotide sequences of HCV and human pegiviruses have been submitted to GenBank and have been assigned accession numbers KU180708 to KU180731.

## RESULTS

### HCV read depths and genome coverage.

**FIG 1:**
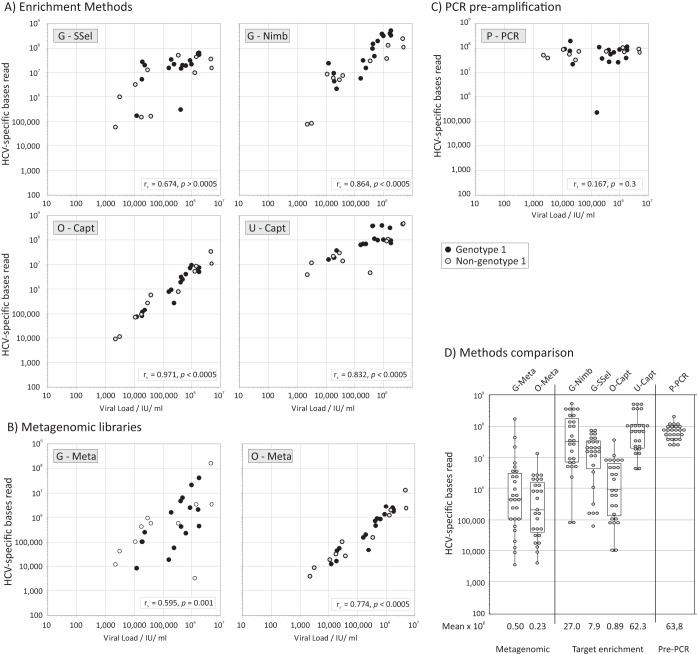
Relationship between viral loads and read counts for each method. (A to C) Total HCV-specific bases read from each sample (*y* axis, log scale) was compared with viral loads separately for target enrichment (A), metagenomic library (B), and sequence preamplified by PCR (C), on a common *x*/*y* scale. Genotype 1 and non-genotype 1 samples are indicated according to the symbol key. The significance of the association between viral loads and read counts was calculated by Spearman's rank order correlation test; Spearman correlation coefficient (*r_s_*) values and *P* values are provided in inset boxes. (D) Distribution of viral loads by method with logarithmic mean values shown below the *x* axis. The box-and-whisker plots shows the median values and 67 and 95 percentiles.

The abilities of different NGS methods to recover HCV sequences from samples with different viral loads was compared (Fig. 1). Each method was effective at detecting HCV sequences in most or all panel samples with a wide range of viral loads, including those as low as 2,000 IU/ml. There was a significant association between read counts and viral loads by using both metagenomic and enrichment methods (Fig. 1), but not after PCR preamplification, where similar read numbers were obtained over a large viral load range (Fig. 1C). Collectively, enrichment consistently recovered more HCV sequence reads than metagenomic methods did (Fig. 1D). There was no evidence for genotype 1 or non-genotype 1 RNA sequences being preferentially detected by any method (*P* > 0.05).

**FIG 2:**
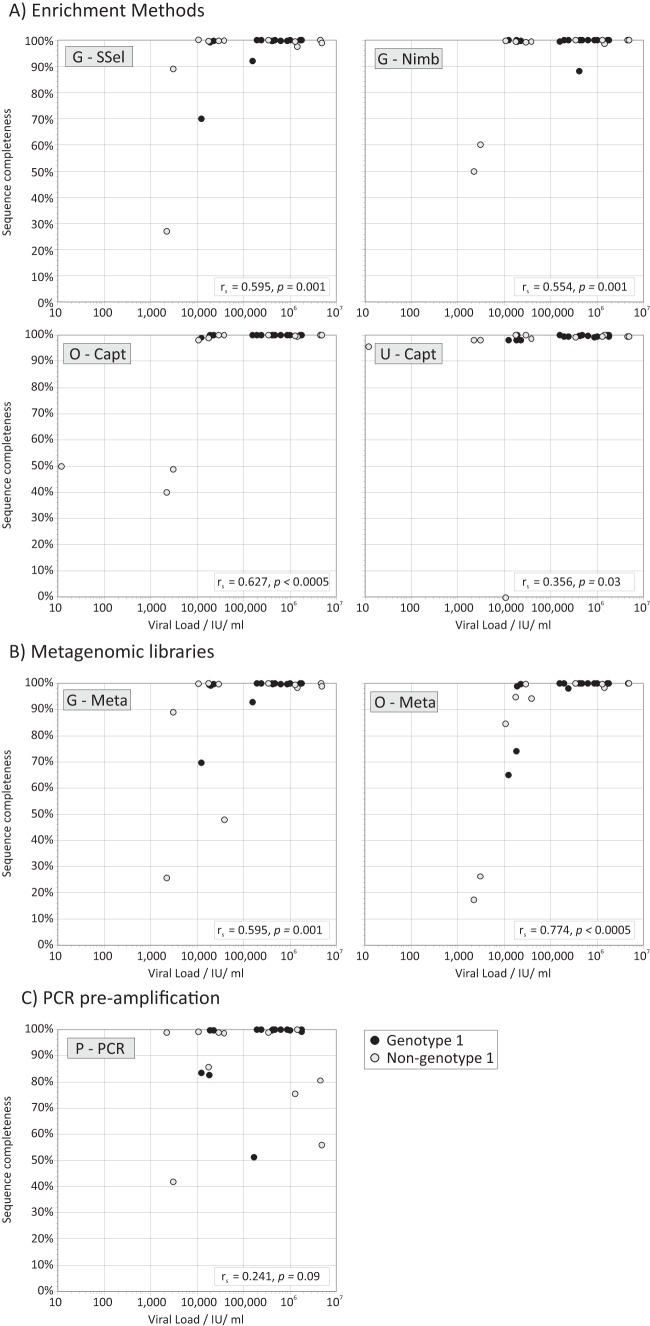
Relationship between viral load and completeness of the HCV consensus sequence from each method. (A to C) The proportion of the whole genome sequenced was compared with viral loads separately for target enrichment (A), metagenomics (B), and sequence preamplified by PCR (C) (plotted on a common *x*/*y* scale). Sequence completeness was expressed as a percentage, assuming a genome length of 9,650 bases. Genotype 1 and non-genotype 1 samples are indicated according to the symbol key. The significance of the association between viral load and genome coverage was calculated by Spearman's rank order correlation test; values of *r_s_* and *P* values are provided in inset boxes.

Reads were assembled by mapping to the closest available reference sequences or *de novo* (Table 1), and a multiple alignment of the assemblies was inferred. Majority rule consensus sequences were inferred from the reads mapped to the assembly and analyzed for completeness. Complete genome sequences (>95% of H77 sequence length) were assembled for the majority of samples by each method (Fig. 2). However, particularly with metagenomic methods, only partial assemblies were generated from samples with lower viral loads. There was no evidence that non-genotype 1 samples were less effectively assembled than genotype 1 samples for all methods (*P* > 0.05).

**FIG 3:**
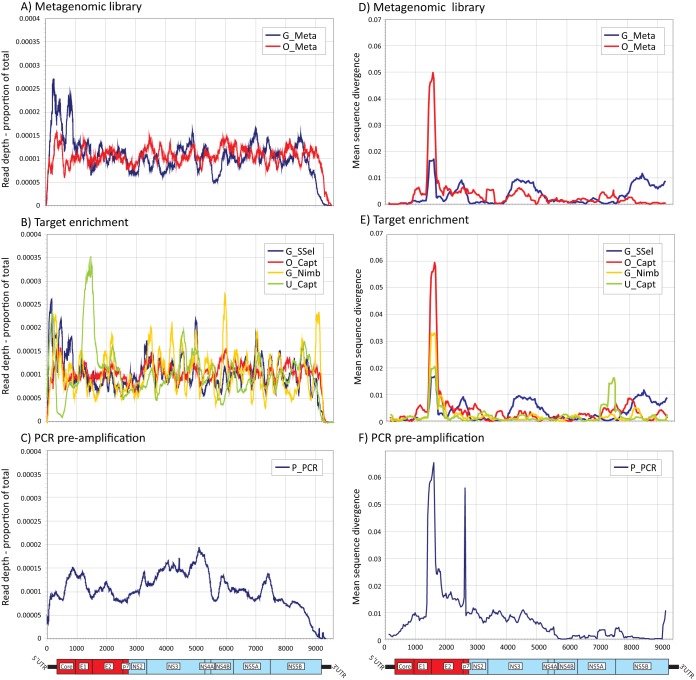
Variability in read depth across the HCV genome coverage and divergence from a global consensus for each of the sequencing methods. (A to C) Mean read coverage across the HCV genome by different NGS methods. Mean coverage was calculated as the number of bases at each site as a proportion of total reads for the sequence (expected mean value of 0.00014); mean values were calculated from samples with >100,000 total reads. Genome positions were based on the H77 reference sequence. A genome diagram of HCV drawn to the same scale as the *x* axis is included below panels A to C. A plot of Z-scores is provided in the supplemental material (see Fig. S1 in the supplemental material). (D to F). Divergence between the global consensus and individual consensus sequences generated by different methods were calculated for a sliding window of 250 bases centered on every 30th base. Mean divergence values for each sequencing method at each site (expressed as proportional distance [*p*-distance]) were plotted for positions homologous to the H77 reference strain. Genomic features of the HCV genome are shown below panels D to F, with structural genes shown in red. A comparable plot of mean values for each genotype is shown in Fig. S3 in the supplemental material.

Inspection of read depth across each consensus sequence showed that each method yielded relatively uniform coverage across the genome (Fig. 3A to C). In general, read depth across the genome was more uniform for the metagenomic methods (Fig. 3A; also see Fig. S1A in the supplemental material, Z-scores ranging from −2 to +2). The lowest coverage for all methods were the 5′ untranslated regions (5′UTRs) and the region beyond the 3′ poly(U) tract (Fig. S2). No sequences were complete at the 5′ and 3′ ends as defined by the sequence span of the H77 sequence. PCR preamplification necessarily limited the coverage of the P-PCR method to positions of the nested sense primer in 5′UTRs and 3′UTRs. Similarly, the reference sequences used for assembly of sequence reads in the G-Meta, G-SSel, and G-Nimb methods lacked the X-tail sequence beyond the polypyrimidine tract and could not be assembled beyond this point. Only the sequences generated by O-Meta and O-Capt were assembled in the highly structured X-tail.

### Accuracy of assembled HCV sequences.

**FIG 4:**
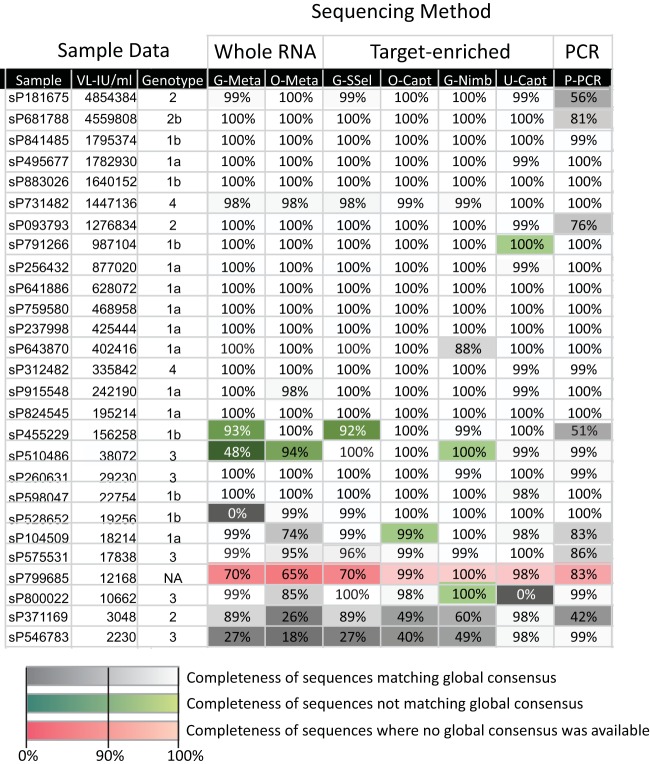
Comparison of the completeness of consensus sequences and their genetic relatedness to each other. Percentage sequence completeness for coding regions is given for each sample. Consensus sequences were assembled from the panel samples by each NGS method and used to define HCV genotype and compared with the genotype identified by conventional genotyping assay (Genotype column). Samples have been ranked by viral load (VL-IU/ml column) (from highest to lowest). Assembled sequences that correspond to the global consensus are shown on a gray/white scale; those that differed by >5% in nucleotide sequence from each other were considered separate strains and are shown on a green scale. Sample sP799685 generated a diverse range of sequences by different NGS methods, and it was not possible to generate a global consensus sequence by combining sequences (red shading). NA, not available.

The genotype of HCV in sequences assembled from each sample was determined by sequence comparisons with reference HCV strains (Fig. 4; also see Table S1 in the supplemental material). Genotype assignments were concordant between NGS methods and the clinical genotyping assays. HCV sequences assembled by NGS were analyzed both by comparison of majority (consensus) sequences and through within-site variability. Majority sequences generated by different NGS methods were generally identical or similar to each other (Fig. 4). However, several assembled sequences failed to match the consensus sequence of other NGS-generated sequences (shown in green) even if NGS defined the same subtype. For one sample (sP799685), four different HCV strains (>5% divergent from each other) were detected by different methods (shown in red). Samples yielding discrepant sequences were typically those with low coverage and lower viral loads (Fig. 4, VL-IU/ml column), particularly the incomplete sequences generated by G-Meta and G-SSel methods. The PCR methodology for HCV genotype 2 in particular frequently failed to generate whole genomes. The following sequences were excluded from further analyses of viral heterogeneity, since the incompleteness of sequence representation precluded generating an informative sample consensus sequence: sP546783, sP371169, sP800022, sP510486, and sP799685.

**FIG 5:**
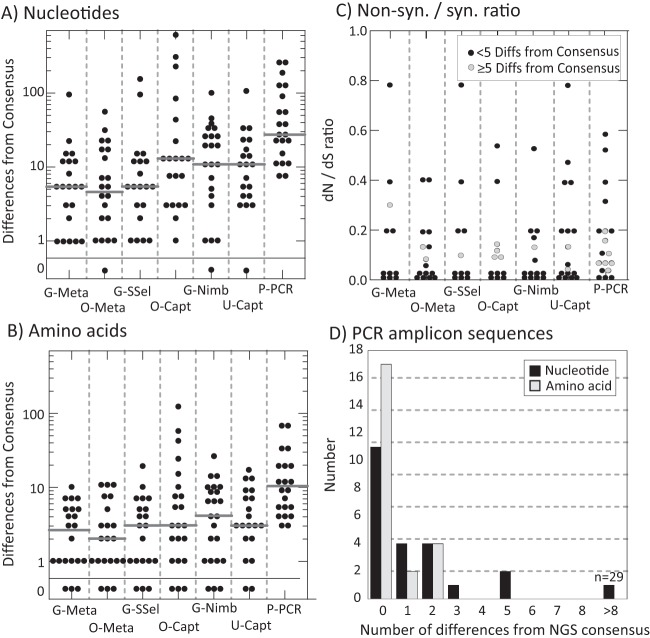
Assessment of viral diversity: sequence differences between the global consensus and majority sequences generated by each NGS method, and the association of HCV viral load with diversity. (A and B) Distribution of the numbers of nucleotide and amino acid differences, respectively (*y* axis, log scale) between the global consensus sequence and the individual majority-rule sequences generated by each NGS method (*x* axis). Sequences phylogenetically unrelated to the global consensus (shaded green in Fig. 4) or where there was no global consensus (shaded red in Fig. 4) have been excluded from this analysis. Gray bars represent median values for the distribution. (C) Nonsynonymous/synonymous ratio of substitutions between each assembled sequence and the corresponding global consensus sequence. More-divergent sequences showing ≥5 differences (Diffs) from the global consensus are plotted with gray filled circles. (D) Distribution of nucleotide and amino acid differences between directly sequenced amplicons derived from the NS3 (positions 3288 to 5727) and NS5B region (positions 7407 to 9366) of 12 samples from the evaluation panel with corresponding regions from the global consensus obtained by NGS methods.

To analyze the similarity of sequences generated by each NGS method, the sequences were compared to a global consensus sequence, representing the combined consensus of the different sequencing methods. Few consensus sequences were identical to global consensus over the whole genome, with many having 10 or more differences (Fig. 5A and B). These differences were concentrated in the hypervariable regions (hypervariable region 1 [HVR1] [E2], 384 to 410; HVR2 (E2), 473 to 480; V3 [NS5A], 2356 to 2379, H77 coordinates [12]) and surrounding E1 and E2 regions (Fig. 3D to F), and divergence was particularly evident for genotype 2 samples (see Fig. S3 in the supplemental material). Different NGS methods showed similar diversity of sequences with the exception of P-PCR, which showed a median of 12 and 9 nucleotide and amino acid differences, respectively, from the global consensus over the complete genome (Fig. 5A and B).

For comparative evaluation of sequencing accuracy with standard PCR/sequencing methodologies, sequences were amplified using separate NS3 and NS5B PCRs (positions 3288 to 5727 and 7407 to 9366, respectively; total 4,00 bases) of genotype 1 panel members (identified in Table S1 in the supplemental material). Sequences directly obtained by Sanger sequencing from the amplicon were compared with the global consensus sequence derived from NGS (Fig. 5D). Most samples showed sequence identity between the two methods. Sequence differences between methods occurred predominantly at polymorphic sites where the base called in the PCR-derived sequence was represented at various proportions among NGS sequences but not called in the majority NGSS consensus sequence (data not shown).

To further determine the accuracy of sequences generated by NGS methods, RNA transcripts of HCV genotypes 1a and 2a were sequenced by representative metagenomic and enrichment methods (O-Meta and O-Capt) to estimate a technical error rate. Sequence errors have originated from misincorporation errors during reverse transcription of the RNA sequences, errors during strand extension during sequencing, and finally bioinformatic errors during base calling and sequence assembly. However, the majority consensus sequences of both transcripts were identical to those of both original clones by the two methods (Table 2), indicating that methods-associated technical errors were not the cause of sequence differences in consensus sequences of the panel samples between methods.

**TABLE 2:** Error rates of representative sequencing methods for HCV genotype 1a and 2a transcripts^*a*^

Method	Transcript^*b*^	Unresolved sites (>5%)^*c*^	Shannon entropy at all sites	Shannon entropy at codon position:		
				1	2	3
O-Meta	1a_AF011751	35	0.0158	0.0150	0.0157	0.0135
	2a_AB047639	42	0.0129	0.0132	0.0143	0.0166
O-Capt	1a_AF011751	25	0.0079	0.0077	0.0075	0.0075
	2a_AB047639	18	0.0065	0.0065	0.0029	0.0032

a All methods had 100% accuracy for the sequence concordance of majority consensus sequence with the sequence of the clone.

b Transcripts are shown by the HCV genotype first and the GenBank accession number.

c Number of ambiguous sites (discordant reads forming >5% of total).

For further evidence that the differences between consensus sequences generated by different methods reflected biological diversity, relative frequencies of synonymous and nonsynonymous substitutions were calculated for the nonstructural gene region (Fig. 5C); natural variability typically occurs at synonymous sites (ratio of nonsynonymous to synonymous evolutionary substitutions [*dN*/*dS* ratio] of ≤0.2 in the HCV genome), while variability arising from technical error associated with the NGS method would be unbiased (*dN*/*dS* ratio of ≈1). All sequences showed *dN*/*dS* ratios below 1, with most ratios substantially lower (<0.2) and consistent with naturally occurring variability. To investigate whether the particularly divergent sequences (≥5 nucleotide differences from the sample consensus) originated from read/assembly errors, they were plotted with a different symbol (gray circles in Fig. 5C). There was little association between the degree of sequence divergence and the *dN*/*dS* ratio.

### Assessment of quasispecies diversity of HCV.

The observed diversity of sequences may originate from naturally occurring variability of variants within samples (“quasispecies”) or technical sequencing errors. The contribution of the latter technical errors to quasispecies diversity was determined though analysis of base counts at each site of sequences derived from the RNA transcripts of genotypes 1a and 2a (Table 2). Analysis of individual base reads at each site revealed that only a small minority of the ≈9,500 sites were polymorphic at the 5% level, ranging from 18 to 42.

**FIG 6:**
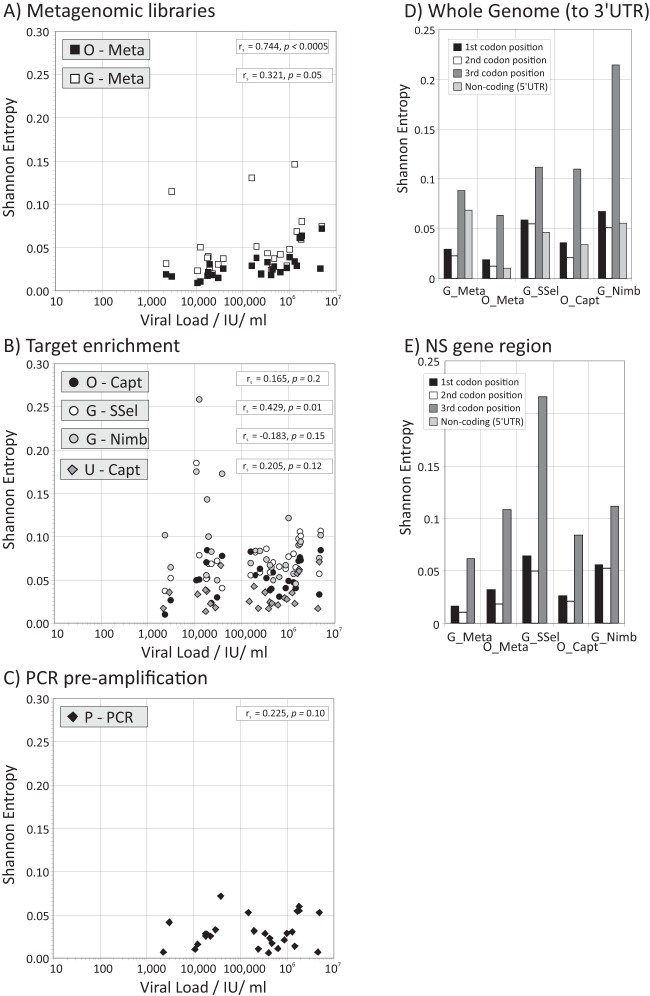
Mean Shannon entropy values of NGS-generated sequences and relationship with viral load. (A to C) Shannon entropy values for polymorphic sites inferred for NGS sequencing methods based on metagenomic libraries (A), target enrichment (B), and PCR preamplification (C). Viral loads are plotted on log scales. (D and E) Shannon entropy values at each codon position in the consensus sequences inferred by each sequencing method based on the whole genome (D) and the nonstructural regions (E).

This diversity was formally quantified through calculation of Shannon entropy where at each nucleotide site, 0 represents no variability, 1 represents equal frequencies of two bases, and 2 represents equal frequencies of all four bases. The mean values for the transcripts (0.0065 to 0.0158) were substantially lower than those observed for RNA sequences present in the panel samples (Table 2; Fig. 6). Variability was evident between sequencing methods, with less diversity observed for pre-PCR or metagenomic sequencing methods. Diversity increased significantly with increasing viral load using metagenomics, but to a much lesser extent with enrichment (Fig. 6).

As with the analysis of sequence differences from the consensus (previous section), within-population variability should be greater at 3rd codon positions (where changes are more likely to be synonymous) if the detected within-site diversity is naturally generated. This was indeed the case, with 2 to 3 times greater Shannon entropy values at 3rd codon positions compared to the 1st and 2nd codon positions both over the whole coding region (Fig. 6D) and in particular if analysis was restricted to the nonstructural gene region (Fig. 6E). As anticipated, no bias toward greater entropy values at 3rd codon positions was evidence in the transcript sequence (Table 2).

### Detection of mixed genotypes.

The ability of different NGS methods to detect coinfections with more than one genotype was determined using a panel of plasma samples containing RNA representing different genotypes in different ratios. These samples included the two mixed-infection plasma samples distributed as part of a United Kingdom national quality control panel (QCMD1 and QCMD2), five plasma samples generated from a mixture of component plasma samples with measured viral loads and calculated ratios (sP731482, sP104509, and JW1 to JW3), a series of RNA transcripts of genotypes 1a and 2a in a wider range of ratios (IVT1 to IVT5 [see Table S1B in the supplemental material]).

**FIG 7:**
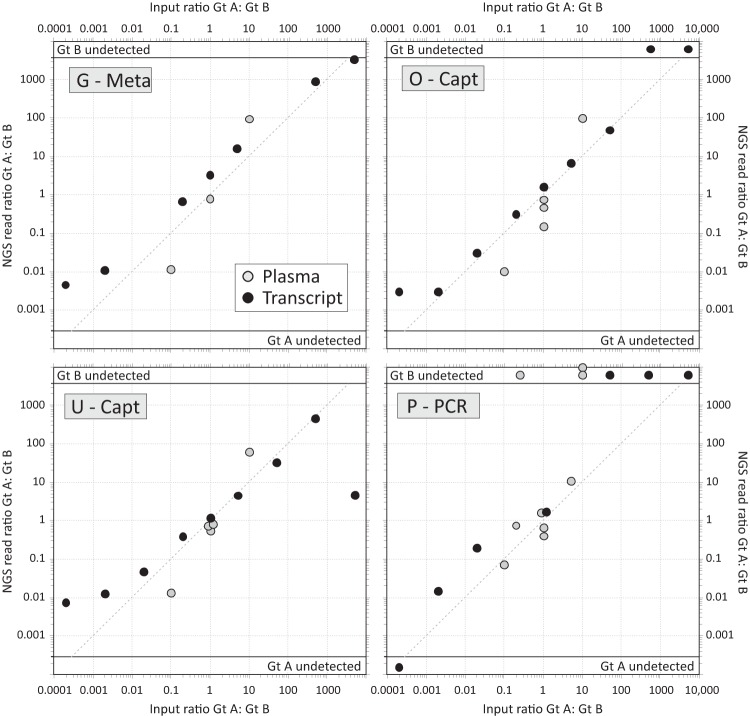
Capacity of NGS to detect mixed-genotype/subtype samples. Observed ratios of NGS read counts between component genotypes genotype A (Gt A) and genotype B (Gt B) (*y* axis) compared to their input ratios (*x* axis), plotted on a log/log scale. The dotted line represents the expected position of data points if the assays were able to detect both input genotypes (genotypes A and B) with equal efficiency. Samples of mixed genotype of known ratio (the input ratio) were acquired from QCMD or through patient samples or *in vitro* transcripts of known genotype that were mixed *in vitro* (listed in Table S1B in the supplemental material).

The reads from each sequencing method were processed using an appropriate bioinformatic pipeline developed by the expert center (Table 1) to infer the genotype and consensus sequence for up to two populations of reads in each sample. The ratios of reads were compared to those of input RNA copies (Fig. 7). For all methods, there was a close and reproducible relationship between the input proportions of plasma and transcript sequences of different genotypes and the relative frequencies of reads by NGS. The majority of observed ratios fell close to the *x* = *y* line added to each graph.

### DAA resistance mutation detection.

**FIG 8:**
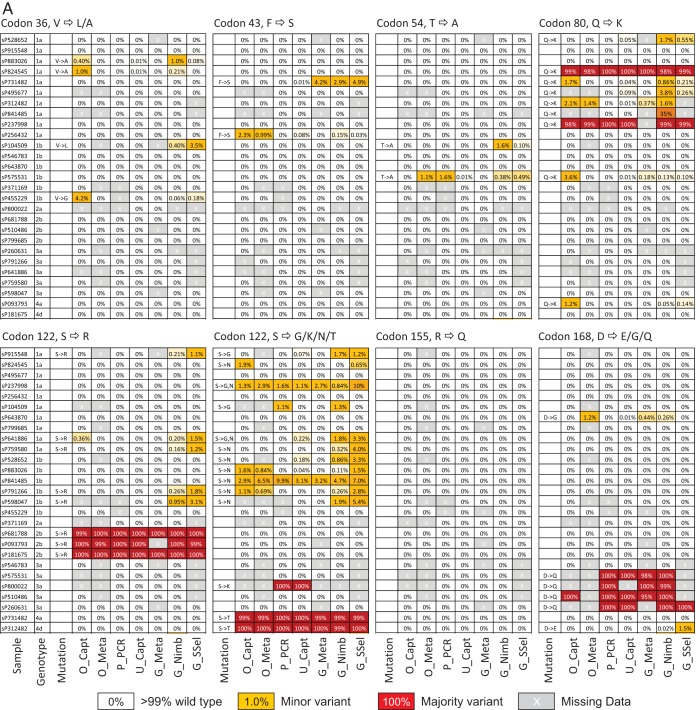

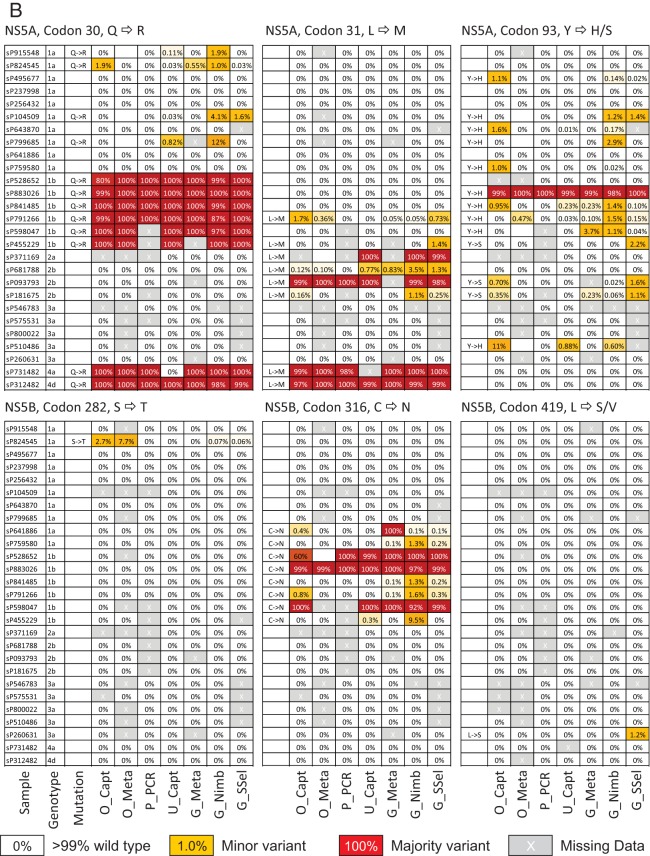
Frequencies of RAVs in the study samples (untreated subjects). Frequencies of resistance-associated mutations in NS3 genes (A) and NS5A and NS5B genes (B) detected by different sequencing methods, shown on a gray or color background to indicate frequencies. Resistance mutations were present either as minor variants (around 1 to 10% of the population; shown by yellow background) or represented the predominant variant in the population (shown by red background). Frequency information from samples with <10 reads at a site were excluded, as were polymorphisms found within a single sequence. Samples have been grouped by genotype.

Frequencies of naturally occurring RAVs in NS3, NS5A, and NS5B genes were compared between the sequencing methods for samples in the evaluation panel; all subjects were DAA treatment naive at the time of sample collection (Fig. 8). Potential RAVs were most frequently detected in the NS3 and NS5A genes, particularly in non-genotype 1 sequences, with highly infrequent detection of resistance at sites associated with inhibitors of the NS5B polymerase (e.g., S282 and L419). Several RAVs were found as majority variants (such as the NS3 Q80K mutation in genotype 1a strains; Fig. 8), and these were consistently detected by different sequencing methods. However, methods varied considerably in their detection of minor populations of RAVs (shown in yellow), with several inconsistencies in their detection or percentage population representations. In general, Glasgow metagenomic and both Glasgow and Oxford capture methods recorded highest frequencies of minor populations of RAVs in all three genes, but in many cases, different polymorphic sites were identified in different samples.

### Coinfecting viruses.

Metagenomic sequence libraries generated by G-Meta and O-Meta were screened by blastn for other human viruses using example sequences obtained from RefSeq (NCBI). Of the 6,783 human viruses screened (NCBI RefSeq viruses r63 [ftp://ftp.ncbi.nlm.nih.gov/refseq/release/viral/]), three samples contained some human pegivirus (HPgV) sequences. One sample, from patient sP104509, could be assembled into a single contig with 100% coverage of the reference and high-read depth (1,600 for O-Meta), and virtually identical assemblies were derived via the O-Meta and G-Meta methods.

## DISCUSSION

Whole-genome sequencing of HCV from clinical samples has until now been considered a costly, laborious, and technically challenging procedure that has not been adopted in routine clinical practice. The major challenge to conventional PCR Sanger sequencing is the inherent diversity of the virus that limits the degree of primer match with different strains and genotypes, generating consensus sequences of limited value and often failing to generate amplicons for large parts of the genome. In contrast, NGS technologies have the potential to generate full-length HCV genomic sequences that enable (i) accurate inference of the full-length, majority consensus HCV genome in the sample and the detection of (ii) minor circulating viral populations within individuals, (iii) mixed-geno(sub)type infections, and (iv) the presence of treatment-associated RAVs along the entire genome. All four metrics will inform future treatment decisions in the new era of DAA therapies.

In order to compare and measure the consistency of different approaches, we evaluated three NGS methodologies, including metagenomic sequencing, target enrichment using both DNA and RNA oligonucleotide probes, and the generation of multiple amplicons by PCR before NGS. For this, we used clinical samples containing a single genotype or a mixture of different genotypes or subtypes across a range of HCV viral loads. All NGS methodologies were able to generate whole genomes from clinical samples and more accurately defined the HCV subtype than the probe-based assay that is commonly used in clinical practice. However, we identified clear advantages and disadvantages to each. The metagenomic approach is fundamentally attractive, since this technique has the capacity to detect other pathogens that may be clinically relevant, and stored metagenomic data can be utilized for viral discovery; as proof of principle, we were able to recover complete HPgV genome sequences in clinical HCV samples using this approach. However, metagenomics provided significantly lower depth of coverage than other methodologies and performed less well at lower HCV viral loads in generating WGS. Furthermore, this approach was relatively costly for the numbers of HCV reads generated, since the vast majority of reads obtained were of human origin and were discarded.

NGS that relies on PCR amplification is currently utilized for the detection of viral resistance. However, in our experience, developing full-length sequences using this approach for even a small number of patients was relatively laborious, requiring multiple PCRs per sample, compared to a single library per sample for metagenomics and the subsequent pooling of 96 libraries in a single tube for sequence capture. It was therefore less suited in its current stage of development for high-throughput analysis. Furthermore, prior knowledge of viral genotype was required; the failure to generate HCV amplicons was particularly evident for HCV genotype 2, since there are currently relatively few complete genome sequences for this genotype available to inform primer design. A linear relationship between HCV viral load and the number of HCV reads was observed with both metagenomic and target enrichment sequencing, but not PCR preamplification where similar numbers of HCV reads were obtained irrespective of viral load. Amplification of viral sequences prior to NGS is therefore likely to be of particular value for samples with low HCV viral loads.

Variability in coverage and sequencing depth across the genome was observed with all methods. This may originate through variability in the degree of match between probe or primer sets to the target viral sequence and therefore differences in the efficiency of target capture or amplification. We have recently shown that introducing probes to better represent known sequence variation can reduce bias in coverage due to probe-target divergence to zero (10). However, incomplete coverage was not a consistent problem for any of the capture methods, which in fact provided substantially greater depth of HCV coverage than metagenomic methods that were probe independent. Capture methods, overall, were better able to generate WGS for the same sequencing effort across a wide range of HCV viral loads.

Overall, there was concordance in the HCV genotypes identified by all NGS methods at each center. While the majority of consensus sequences obtained by each sequencing center were identical to each other, unrelated sequences were obtained in a minority of cases; this could be explained by sequencing error, cross-contamination, or preferential sequencing of one strain over another in samples from patients with mixed-strain infection. Sequencing error was considered unlikely to have contributed significantly to these differences, as the *dN*/*dS* ratio was consistently low with an increase in variability at the 3rd codon site (usually a synonymous position) in keeping with natural occurring variability. Furthermore, NGS of RNA transcripts demonstrated extremely low frequencies of sequencing errors from a defined template, while NGS-derived sequences differed little from Sanger-sequenced amplicons from the NS3 and NS5A/5B regions (Fig. 5D).

Infection with mixed HCV genotypes has been frequently reported (13–15), but its true incidence is unknown, since existing genotyping assays are not designed to assess this. The impact on clinical care of mixed-genotype infections is not yet clear, but theoretically, more drug-resistant genotypes such as genotype 3 could result in treatment failure as a result of emerging dominance during treatment (16). NGS methodologies that routinely captured this data would therefore represent an important advance. For all methodologies, we demonstrated that NGS was remarkably accurate in determining the ratio of mixed genotypes in clinical samples. In addition to genotyping, we assessed the presence or absence of resistance mutations within NS3, NS5A, and NS5B using each sequencing method. Majority variants were reliably detected by all methods, but variation was noted in minority variant detection. Both these data and the mixed-genotype experimental data suggest that the detection of minority variants is less reliable at lower ratios.

In summary, we provide a comprehensive analysis of three NGS sequencing methodologies for the generation of full-length HCV genomes. Our data suggest that HCV target enrichment is highly effective, suitable for high-throughput analysis, and relatively effective at low viral loads, generating deep coverage along the HCV genome. The metagenomic approach remains attractive because the libraries generated may be probed for additional pathogens that may contribute to disease development and which will provide a rich data set for future research endeavors in pathogen discovery. PCR preamplification is relatively laborious but may still have a role in samples with very low viral loads. We have shown that WGS of HCV is readily achievable across multiple sites in the United Kingdom. In the era of DAA therapy, where a single course of therapy routinely costs >£30,000, we believe that NGS for the generation of WGS that accurately defines viral genotype, and readily detects both RAVs and mixed infections should be routinely employed. Sequencing by any of the methods evaluated in the current study can be achieved at a cost of approximately £120/sample, comparable to that of the existing clinical genotyping assays. The generation of WGS for HCV nationwide would be hugely informative, guiding clinical practice while concurrently providing an invaluable data set for epidemiology studies and future research.

## Supplementary Material

Supplemental material
