# Supplementary material for: Comparison of Next-Generation Sequencing Technologies for Comprehensive Assessment of Full-Length Hepatitis C Viral Genomes
Source: J Clin Microbiol. 2016 Sep 23;54(10):2470–84. doi: 10.1128/JCM.00330-16 (PMC5035407; doi:10.1128/JCM.00330-16)
Supplement: Supplemental material [file supp_54_10_2470__index.html]

Supplemental material 

# Comparison of Next-Generation Sequencing Technologies for Comprehensive Assessment of Full-Length Hepatitis C Viral Genomes

## Supplemental material

- Supplemental file 1 -

  Tables S1 (HCV genotypes and subtypes in evaluation panel) and S2 (Primers used for sequencing of NS3 and NS5B amplicons) and Fig. S1 (Read depths across the genome for different NGS methods), S2 (Completeness of consensus sequences at the 5′ and 3′ ends), and S3 (Sequence divergence between the global consensus and individual sequences of different genotypes generated by NGS)

  PDF, 1.4M
