# Supplementary material for: Comparison of Next-Generation Sequencing Technologies for Comprehensive Assessment of Full-Length Hepatitis C Viral Genomes
Source: J Clin Microbiol. 2016 Sep 23;54(10):2470–84. doi: 10.1128/JCM.00330-16 (PMC5035407; doi:10.1128/JCM.00330-16)
Supplement: Supplemental material [file JCM.00330-16_zjm999095136so1.pdf]

Supplementary Data

Table S1: HCV genotypes and subtypes in the evaluation panel

A) Genotype and viral loads of *single genotype samples*

Sample	VL[1] - IU/ml	Genotype[2]	G-Meta	O-Meta	G-SSel	O-Capt	G-Nimb	U-Capt	P-PCR	Ampl.[3]
sP528652	19,256	1b	1b	1b	1b	1b	1b	1b	1b	1b
sP915548	242,190	1a	1a[3]	1a	1a	1a	1a	1a	1a	1a
sP883026	1,640,152	1b	1b	1b	1b	1b	1b	1b	1b	1b
sP824545	195,214	1a	1a	1a	1a	1a	1a	1a	1a	1a
sP731482	1,447,136	4	4a	4a	4a	4a	4a	4a	4a	
sP495677	1,782,930	1a	1a	1a	1a	1a	1a	1a	1a	1a
sP312482	335,842	4	4d	4d	4d	4d	4d	4d	4d	
sP841485	1,795,374	1b	1b	1b	1b	1b	1b	1b	1b	
sP237998	425,444	1a	1a	1a	1a	1a	1a	1a	1a	1a
sP256432	877,020	1a	1a	1a	1a	1a	1a	1a	1a	1a
sP104509	18,214	1a	1a	1a	1a	1a	1a	1a	1a	
sP546783	2,230	3	3a	3a	3a	3a	3[4]	3a	3a	
sP643870	402,416	1a	1a	1a	1a	1a	1a	1a	1a	1a
sP575531	17,838	3	3a	3a	3a	3a	3a	3a	3a	
sP371169	3,048	2	2a	2a	2a	2a	2a	2a	2a	
sP800022	10,662	3	3a	3a	3a	3[2]	3a	ND[5]	3a	
sP681788	4,559,808	2b	2b[3]	2b	2b	2b	2b	2b	2b	
sP510486	38,072	3	3a	3a	3a	3a	3a	3a	3a	
sP799685	12,168	1a	1a	1a	1a	1a	1a	1a	1a	
sP260631	29,230	3	3a	3a	3a	3a	3a	3a	3a	
sP791266	987,104	1b	1b	1b	1b	1b	1b	1b	1b	1b
sP641886	628,072	1a	1a	1a	1a	1a	1a	1a	1a	1a
sP759580	468,958	1a	1a	1a	1a	1a	1a	1a	1a	1a
sP598047	22,754	1b	1b	1b	1b	1b	1b	1b	1b	1b
sP093793	1,276,834	2	2b	2b	2b	2b	2b	2b	2b	
sP181675	4,854,384	2	2b	2b	2b	2b	2b	2b	2b	
sP455229	156,258	1b	1b	1b	1b	1b	1b	1b	ND[5]	

[1]VL: viral load

[2]Genotype provided by referring laboratories

[3]Sample identification and genotype/subtype identified by amplicon sequencing of NS3 and NS5 regions by PCR at PH

[4]Subtype unidentifiable

[5]Not done – no sequence assembled

B) Genotype ratio of mixed genotype/subtype transcripts and samples

Sample	Type	GtA:GtB	Ratio
HCVG10-02	Plasma	1b:3a	≈1:1
HCVG10-04	Plasma	3a:5a	≈1:1
JW1	Plasma	1a:3a	1:10
JW2	Plasma	1a:3a	10:1
JW3	Plasma	1a:3a	1:1
sS786324	Plasma	1a:1b	1:4
sS589393	Plasma	4d:1b	10:1
IVT1	RNA transcript	1a:2a	1:1
IVT2	RNA transcript	1a:2a	1:5
IVT3	RNA transcript	1a:2a	5:1
IVT4	RNA transcript	1a:2a	1:50
IVT5	RNA transcript	1a:2a	50:1
IVT6	RNA transcript	1a:2a	1:500
IVT7	RNA transcript	1a:2a	500:1
IVT8	RNA transcript	1a:2a	1:5000
IVT9	RNA transcript	1a:2a	5000:1

TABLE S2

Primers used for sequencing of NS3 and NS5B amplicons

Genotype	Gene	Primer name	Primer sequence (5' to 3')
1a	NS34A	1a34a-Seq5'3298	ATG GAG ACC AAG CTC ATC ACG TG
1a	NS34A	1a34a-Seq5'3763	ACG AGG CAC GCC GAT GTC ATT CC
1a	NS34A	1a34A-Seq5'3922	CGT GGA GTG GCT AAG GCG GTG GA
1a	NS34A	1a34a-Seq5'4344	GCA TCG GCA CTG TCC TTG ACC
1a	NS34A	1a34a-Seq5'4762	GAC CCT ACC TTT ACC ATT GAG AC
1a	NS34A	1a34a-Seq5'5167	ATC GTG GGA TCA GAT GTG GA
1a	NS34A	1a34a-Seq3'3742	GAA TGA CAT CGG CGT GCC TCG
1a	NS34A	1a34a-Seq3'3964	GAG GAG TTG TCC GTG AAC ACC G
1a	NS34A	1a34a-Seq3'4221	AGG AAC TTG CCG TAG GTG GA
1a	NS34A	1a34a-Seq3'4605	CTG GTC GGG ATG ACA GAC ACG TC
1a	NS34A	1a34a-Seq3'5107	TAG TGC ACA CGG TGG CTT GGT
1a	NS34A	1a34a-Seq3'5517	AGG GCC TTC TGC TTG AAC TGC TC
1a	NS5B	1a5b-Seq5'7567	ACG GGT CAT GGT CGA CGG TCA G
1a	NS5B	1a5b-Seq5'7983	CA CAT CAA CTC CGT GTG GAA AGA CCT TCT GG
1a	NS5B	1a5b-Seq5'8307	CCA CAG TCA CTG AGA GCG ACA TCC GTA CGG
1a	NS5B	1a5b-Seq5'8695	GAC TTG GAG CTC ATA ACA TC
1a	NS5B	1a5b-Seq5'9109	CGC CCT TGC GAG CTT GGA GAC ACC G
1a	NS5B	1a5b-Seq3'7959	TCC AGA AGG TCT TTC CAC ACG GAG TT
1a	NS5B	1a5b-Seq3'8300	GGT AAA TTG CCT CCT CCG TAC GG
1a	NS5B	1a5b-Seq3'8625	GGG GCG GAG TAC CTG GTC ATA GC
1a	NS5B	1a5b-Seq3'8950	GTG GTT CTA TGG AGT AGC AGG
1a	NS5B	1a5b-Seq3'8978	TGG AGT CTT TGA ATG ATT GGA GGT
1a	NS5B	1a5b-Seq5'7558	GAT CTC AGC GAC GGG TCA TGG TC
1a	NS5B	1a5b-Seq3'9355	TCG GTT GGG GAG GAG GTA GA
1a	NS5B	1a5B-seq5'8686	CAG AAT ACG ACT TGG AGC T
1a	NS5B	1a5B-seq3'8995	GCG CTG AGG CCG TGG AGT C
1b	NS34A	1b34a-Seq5'5396	GCG TGG TCA TTG TGG GCA GG
1b	NS34A	1b34a-Seq5'5180	ATG TGG AAG TGT CTC ATA CGG
1b	NS34A	1b34a-Seq5'4621	GGT CTT GAT GTG TCC GTC ATA CC
1b	NS34A	1b34a-Seq5'4201	GAA CTG GGG TAA GGA CCA TCA CCA C
1b	NS34A	1b34a-Seq5'3673	GTA GAC CAG GAC CTC GTC GGC TG

1b	NS34A	1b34a-Seq5'3388	GAG ATA CTT CTG GGA CCG GCC GA
1b	NS34A	1b34a-Seq3'5481	CCT GTT CGA TGT AAG GGA GGT G
1b	NS34A	1b34a-Seq3'5073	AGG TAG GGG AAG TTG TCT CCT GCC TG
1b	NS34A	1b34a-Seq3'4751	GGC ACG GTC GTC GTC TCA ATG GTG
1b	NS34A	1b34a-Seq3'4409	CTC GAT GTT GGG ATG TGG CAC G
1b	NS34A	1b34a-Seq3'3999	CCA CTT GGA ATG TCT GCG G
1b	NS34A	1b34a-Seq3'3641	GAG GTC CTG GTC TAC ATT GGT GTA C
1b	NS5B	1b5b-Seq5'7576	TCT TGG TCT ACC GTG AGC GAG GAG GC
1b	NS5B	1b5b-Seq5'7590	GAG GAG GCy AGT GAG GAC GTC G
1b	NS5B	1b5b-Seq5'7981	CTC CGT GTG GAA GGA CTT GCT GGA
1b	NS5B	1b5b-Seq5'8330	GAG TCA ATC TAC CAA TGT TGT GAC
1b	NS5B	1b5b-Seq5'8629	CGA GTC TTC ACG GAG GCT ATG AC
1b	NS5B	1b5b-Seq5'9021	CTT AGC GCA TTT TCA CTC C
1b	NS5B	1b5b-Seq3'7960	GTC TTC CAG CAA GTC CTT CCA CAC GG
1b	NS5B	1b5b-Seq3'8153	CCG TAT GAG GAG CCC ATC ACG
1b	NS5B	1b5b-Seq3'8617	GAG TAC CTA GTC ATA GCC TCC G
1b	NS5B	1b5b-Seq3'8973	GTC GTT GAA TGA TCT GAG GTA
1b	NS5B	1b5b-Seq3'9204	ACG CAG CCG GGA TTG GAG TGA G
1b	NS5B	1b5b-Seq3'9320	ACA GAA AGT AGG AGT AGG CAC

Figure S1: Read depths (Z-scores) across the genome for different NGS methods

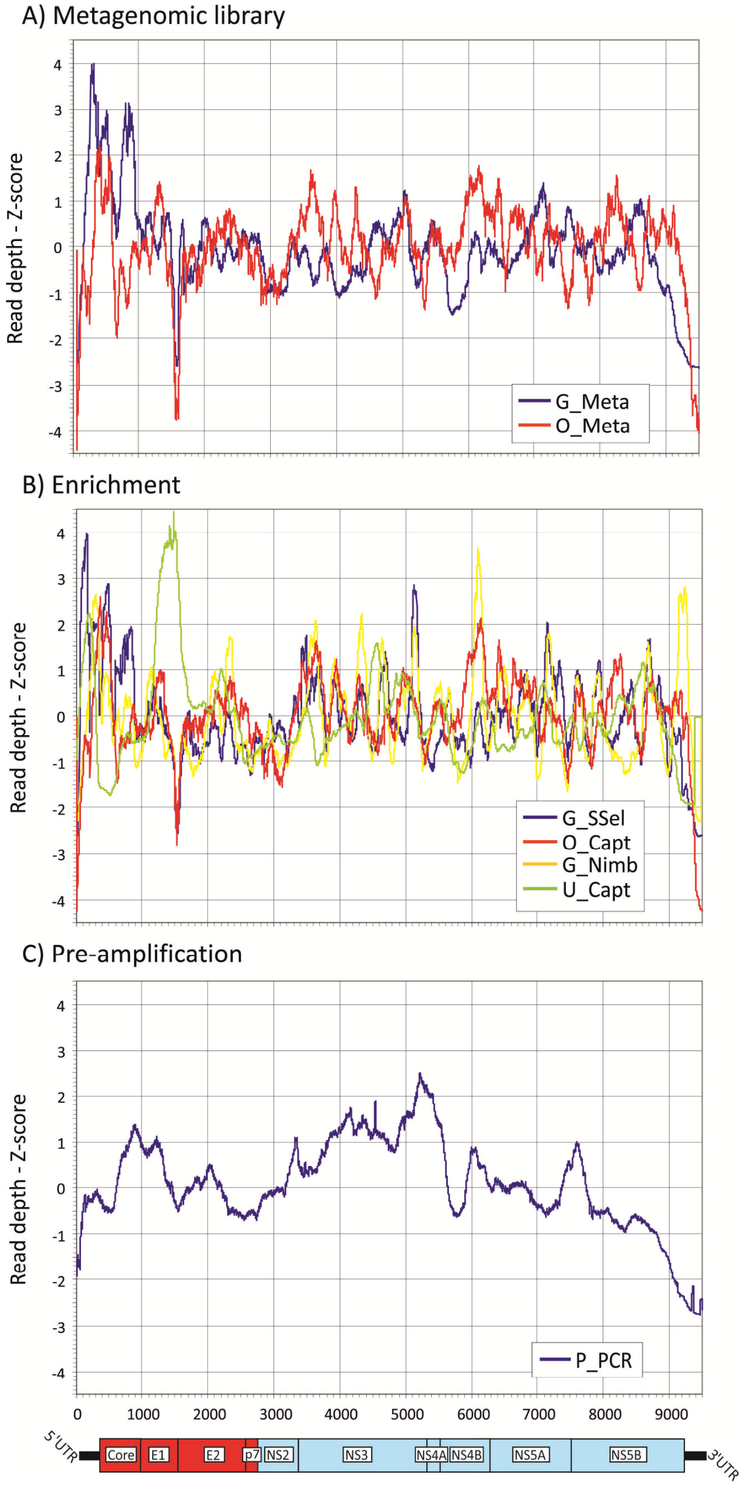

Z-scores of mean coverage across the HCV genome by different NGS methods. Genome positions were based on the H77 reference sequence. A genome diagram of HCV drawn to the same scale as the x-axis is included below.

Figure S2: Completeness of the consensus sequences at the 5' and 3' ends

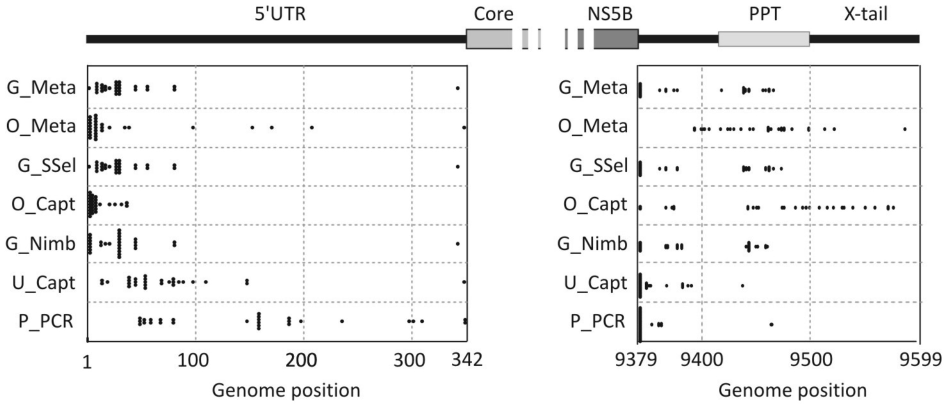

Position of 5' and 3' ends of sequences assembled by different NGS methods, where dots represent termination points for each assembled sequence. For orientation, genome diagrams of the 5'UTR and 3'UTR are shown above. PCR pre-amplification necessarily limited the coverage of the P-PCR method to positions of the nested sense primer in 5' UTRs at 63 (G1a), 169 (G1b and G2), 190 (G3a) and 312 (G4) and anti-sense primer in 3' UTRs at 9395 (G1a), 9370 (G1b), 9295 (G3a) and 9290 (G4). Similarly, the reference sequences used for assembly of sequence reads in the G-Meta, G-SSel and G-Nimb lacked the X-tail sequence beyond the poly-pyrimidine tract (PPT) and could not be assembled beyond this point.

Figure S3: Sequence divergence between the global consensus and individual sequences of different genotypes generated by NGS

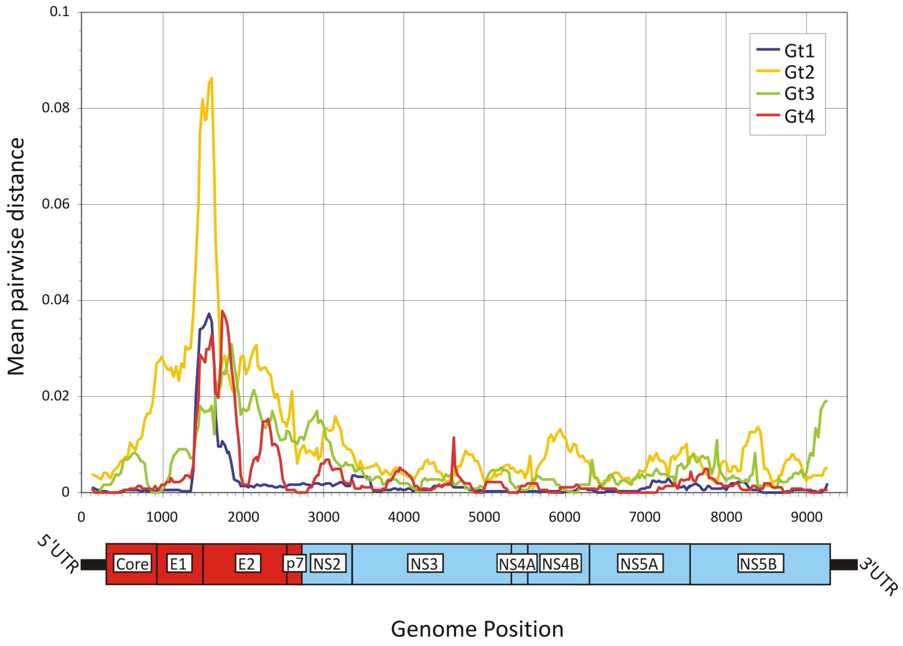

Divergence values were calculated for a sliding window of 250 bases centred on every 30th base. Mean values for sample genotype were plotted for positions homologous to the H77 reference strain. Genomic features of the HCV genome are shown below, with structural genes shown in red.
